# Intermittent or Sequential Topical Tacrolimus in Atopic Dermatitis: Systematic Review and Meta-Analysis

**DOI:** 10.7759/cureus.50640

**Published:** 2023-12-16

**Authors:** Sandipan Dhar, Abhishek De, Abhijit Saha, Kruttika R Chitnis, Abhishek Mane, Dhiraj Dhoot, Hanmant Barkate

**Affiliations:** 1 Dermatology, Institute of Child Health, Kolkata, IND; 2 Dermatology, Calcutta National Medical College and Hospital, Kolkata, IND; 3 Pharmacology and Therapeutics, Seth Gordhandas Sunderdas Medical College and King Edward Memorial Hospital, Mumbai, IND; 4 Global Medical Affairs, Glenmark Pharmaceuticals Limited, Mumbai, IND

**Keywords:** immunomodulator, pruritus, eczema, topical calcineurin inhibitors, immunodermatology

## Abstract

Topical calcineurin inhibitors (TCIs) and topical corticosteroids (TCS) are the mainstays of flare management for atopic eczema or atopic dermatitis (AD). Tacrolimus (an immunomodulator), belongs to the class of calcineurin inhibitors, with promising efficacy in AD. We performed this systematic review to obtain an up-to-date coverage map of controlled clinical trials of sequential or intermittent treatments with TCI as a therapeutic intervention for AD. Articles of interest were retrieved from PubMed, Google Scholar, and EMBASE published between between January 2000 and March 2023. Key words were “calcineurin inhibitors,” "corticosteroids,” "atopic dermatitis,” "pruritus,” "sequential,” "intermittent" and "consecutive" while fixed language search consisted of "Intermittent topical calcineurin inhibitors AND topical corticosteroids AND atopic dermatitis OR eczema" AD patients who were administered sequential and/or intermittent applications of TCI for management of atopic eczema were included. Outcome measures included but were not limited to Scoring of Atopic Dermatitis (SCORAD) and the Eczema Area Severity Score (EASI). Four clinical trials were considered for the purpose of review. A total of 101 patients with AD were analysed. The risk of bias was low in two studies, while the other two had an unclear risk of bias. Overall, pooled data from two trials revealed that sequential therapy with TCS/TCI was comparable to monotherapy or emollients, as the test for overall effect determined was non-significant with a p-value of 0.33. The two studies were highly heterogeneous, as indicated by a very high I^2^ of 92% and an extremely significant p-value (p=0.0005). Sequential therapy with TCS and TCIs was effective and well tolerated in the management of AD and it may be considered an important treatment approach during the initial period.

## Introduction and background

Atopic dermatitis (AD) is a chronic, recurrent, inflammatory skin disease characterized by intense pruritus (itch) and eczematous lesions [1]. AD affects about 20% of children and 10% of adults globally and a rising prevalence has been observed worldwide [2], including Asia [3]. It has a significant burden on the quality of life of the patients, families, and caregivers which leads to increased work-related impairment as well as healthcare costs [4,5]. It is also one of the most common dermatoses encountered in the paediatric population in India, yet our knowledge and perception about the nature and character of the disease are mostly acquired from Western literature [6]. The two most important factors implicated in the pathogenesis of the disease, viz. ‘genetic’ and ‘environmental’ vary widely from country to country and place to place, and the clinical patterns of disease manifestations also vary significantly [6]. Sarkar et al., in a study of clinical epidemiological profiles and factors affecting the severity of AD in North Indian children, highlighted that differences in climatic conditions, dietary habits, prolonged breastfeeding, late weaning, larger families, and low frequency of personal and family history of atopy could be contributing factors to the milder form of AD in children [7]. The aetiology of AD is multifactorial, involving a complex interplay of genetics, immunological, and environmental factors, leading to immune regulation and skin barrier dysfunction [8].

Acute exacerbations in the form of flares are an integral part of the AD course and flares are generally defined as disease worsening, requiring escalation/intensification of treatment [8]. Various flare-triggering factors; including endogenous and environmental factors, modulate the intensity and frequency of the flares [9]. Restoration of skin barrier integrity with regular emollient use and prompt topical anti-inflammatory therapy either with topical corticosteroids (TCS) or topical calcineurin inhibitors (TCIs) form the mainstay of clinical management of flares in AD, whereas systemic therapy is considered for moderate-to-severe disease [9]. The International Study of Life with Atopic Eczema (ISOLTE) trial showed that patients with moderate-to-severe disease, spend an average of one in three days with flare (average of nine flares per year lasting 15 days each time) [9]. In India, where we mostly come across mild to moderate forms of AD, topical treatment forms the sheet anchor of its management [10].

The flare-control approach can be reactive or proactive. A reactive approach involves daily moisturizer administration to improve skin hydration and alleviate epidermal barrier dysfunction as part of a maintenance treatment plan, with anti-inflammatory therapies reintroduced after disease flares. A proactive approach involves the long-term, intermittent application of topical corticosteroids or topical calcineurin inhibitors to previously and newly affected areas of the skin, along with maintenance treatment with emollients to unaffected areas. Nevertheless, flare control is challenging, especially when considering treatment-resistant presentations of AD [9]. Moreover, the potential adverse drug reactions, such as skin atrophy, purpura, telangiectasia, striae, hypopigmentation, and acneiform eruptions that are associated with prolonged use of TCS [9,11] paved the way for TCIs, such as tacrolimus, as a useful steroid-sparing molecule in the armamentarium for the long-term control of the disease [12]. 

Intermittent dosing with the potent TCS and/or combination therapy with TCS and TCI has frequently been used in routine management of AD to overcome the problems accompanying the long-term use of TCS [13]. These therapeutics developed with the recognition of the heterogeneity of disease could enable a precision medicine approach to optimize patient outcomes in AD. However, a systematic basis for these treatments remains lacking. The objectives of this systematic review were to produce an up-to-date coverage map of controlled clinical trials of sequential or intermittent treatments with a TCI as one of the agents for the management of AD and to perform a meta-analysis of available RCT evidence that could assist in making treatment recommendations by summarizing the available evidence using qualitative and quantitative methods.

## Review

Material and methods

Protocol and Reporting

**Figure 1:**
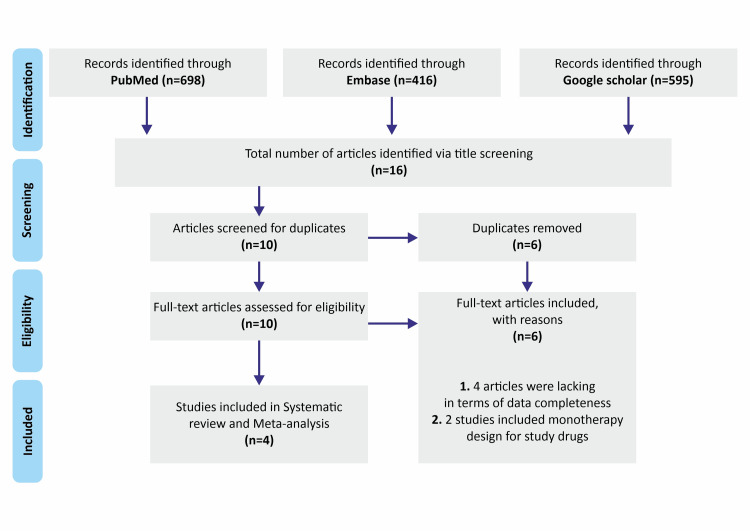
Study identification and selection

This study was conducted in accordance with the Preferred Reporting Items for Systematic Reviews and Meta-Analyses (PRISMA) guidelines. The protocol for this systematic review and meta-analysis was registered with PROSPERO (Reg. no: CRD42023409445). The total number of studies identified via different databases, number of studies considered for full-text evaluation, and reasons for exclusion are illustrated in Figure 1.

Systematic Literature Review

Searches were conducted in PubMed, Google Scholar, and EMBASE to identify English language articles published between January 2000 and March 2023 reporting clinical trials for the evaluation of possible treatments for patients with AD. The search strategies included a combination of controlled vocabulary terms, free-text search terms for diseases, and study designs of interest. Additionally, we manually searched the bibliographies of the included studies. The search strings used for the identification of studies from PubMed and Google Scholar were as follows: topical calcineurin inhibitors and topical corticosteroids in atopic dermatitis or atopic eczema, and a filter to identify all published clinical trials (randomized and non-randomized) focusing on sequential topical therapy or intermittent topical treatments for AD, based on our inclusion criteria. Free text search consisted of the following words: “topical calcineurin inhibitors", "topical corticosteroids", "atopic dermatitis", "pruritus", "sequential", "intermittent" and "consecutive". They were used along with the Boolean operators “AND” or “OR” between them. The fixed language search used in EMBASE consisted of "Intermittent topical calcineurin inhibitors AND topical corticosteroids AND atopic dermatitis OR eczema" filtered by trials and publication date. The titles and abstracts of the articles yielded by this search were screened by independent reviewers to determine whether they met the inclusion criteria.

Identification and Selection of Studies

An elaborate search was conducted to evaluate the effects of sequential treatment with topical corticosteroids and calcineurin inhibitors in the management of AD. However, to the best of our knowledge, we did not come across any such guidance document standardizing the terminology of sequential use of these drugs for management of patients of AD. Trials that met the PICOS (patients, intervention, comparator, outcome, study in) meta-analysis. (1) Patients: physician diagnosed with paediatric or adult atopic dermatitis. (2) Intervention: sequential and/or intermittent application of topical calcineurin inhibitors and topical corticosteroids for the management of AD; (3) comparator: treatment modality other than sequential and/or intermittent application of topical calcineurin inhibitors and topical corticosteroids or sequential and/or intermittent application of topical corticosteroids and any treatment modality other than topical calcineurin inhibitors for the management of AD; (4) AD evaluated by one of the following outcomes: Eczema Area and Severity Index score, Investigators’ Global Assessment, severity of pruritus and sleep disturbance scores, QOL evaluation, SCORing Atopic Dermatitis (SCORAD), lesions evaluated by the three major symptoms of erythema/acute papules, exudation/crusts/scratching, lichenification/chronic papules, as recommended by the Japanese Dermatological Association, and adverse events. Exclusion criteria were studies where topical calcineurin inhibitors were used as monotherapy or in a non-sequential manner in both comparator arms. Studies with insufficient outcome data were also excluded as well as narrative/systematic reviews and meta-analysis, preclinical studies, and letters to the editor on the management of atopic dermatitis. Finally, all qualifying articles available in English were chosen for review.

Data extraction

Data on the patient population, treatments, time points, and available outcomes of interest were extracted by two investigators and validated for accuracy by a third senior investigator. Two reviewers independently used a standard data extraction form to retrieve relevant data from articles. The extracted data included the author, country, study design, sample size, publishing date, age, and AD classification. The primary endpoint was a reduction in AD scores or symptomatology as per the standard clinical assessments mentioned in the eligibility criteria.

Any disagreement over whether to include specific criteria with respect to efficacy parameters (erythema, burning, pruritus, and scoring) was settled by discussion or, if required, arbitration by a third reviewer. We collected and analysed information on the effectiveness of a sequential treatment regimen based on topical calcineurin inhibitors or topical corticosteroids as opposed to monotherapy with either of the two drugs.

Risk of Bias Assessment

The risk of bias evaluation was performed separately for each study by two reviewers in a blinded fashion. Two authors independently assessed the quality of the included studies based on the Cochrane Handbook for Systematic Reviews of Interventions. Using the robvis visualization tool, the ‘‘risk-of-bias’’ table was constructed based on the following domains: details on methods of random sequence generation, incomplete outcome data, selective outcome reporting, and other bias. The overall quality of each study was evaluated as ‘‘low risk of bias, high risk of bias,’’ or ‘‘unclear”.

Data Synthesis and Statistical Analysis

All included studies were included in the qualitative review and systematic evaluation to summarize the key findings. The meta-analysis was carried out with the help of the RevMan software version 5.3. Only two of the included studies were eligible for conducting the meta-analysis because there was a paucity of studies with respect to the inclusion criteria. Statistical heterogeneity among the included studies was assessed using ‘p’ and ‘I2’. When I2< 50% and p>0.1, a fixed-effects model was applied; otherwise, a random-effects model was used. The inverse variance and Mantel-Haenszel methods were used to combine separate statistics. p<0.05. Due to the potential heterogeneity across the included studies, the data were pooled using a random-effects model to account for any differences between them.

Results

Systematic Review of Literature

Study selection and characteristics: A thorough literature search gave us 1709 studies from all databases. Following title screening and duplicate removal, the total number of studies was reduced to 10. A full-text review of the selected articles further reduced the number of articles to four. Of these four studies, two studies (Takeuchi et al.* *[14] and Nakahara et al.* *[15]) were randomized controlled trials and were considered for pooled analysis for assessment of clinical benefit with respect to sequential and monotherapy, while the remaining two studies (Kubota et al*. *[16] and Keaney et al*. *[17]) were open-label pilot studies included in the qualitative analysis of the patient response to sequential therapy with TCI and TCS, along with the other two studies. The key findings are summarized in Table 1.

**Table 1:** Details of study design, treatment interventions, analysis and inference AD: atopic dermatitis, TCS: topical corticosteroids, TCI: topical calcineurin inhibitors, QOL: quality of life

Study name	Study design	Drugs used in sequential therapy	Efficacy analysis	Safety analysis	Study result	Inference
Takeuchi S et al. [14]	Two-group parallel assignment: patients who received topical tacrolimus monotherapy as maintenance therapy after induction therapy and patients who received emollient only for maintenance therapy after induction therapy	Oral tacrolimus, topical tacrolimus	Anti-pruritic efficacy	Not assessed	Two-thirds of patients (44/68; 64.7%) showed relief from pruritus after induction therapy. Pruritus recurred in 23.8% (5/21) of the tacrolimus monotherapy group and in 100% (21/21) of the emollient group during maintenance period, a difference that was statistically significant.	Use of topical tacrolimus is effective in controlling pruritus of AD compared to emollient.
Nakahara T et al. [15]	Open-label comparison between right and left half in the same patient	0.05% betamethasone butyrate propionate ointment, 0.1% tacrolimus ointment, emollients	eruption score reduction: A: erythema/acute papules, B: exudation/crusts/scratching, C: lichenification/ chronic papules.	No adverse effects	Lichenification/chronic papules: eruption score reduction statistically significant between	The intermittent topical steroid/tacrolimus sequential therapy may be a useful adjunctive treatment for AD.
Kubota et al. [16]	The study regimen consisted of 3 phases. Induction phase: 2-week period with application of 0.03% tacrolimus ointment in the morning and application of a strong- or weak-potency corticosteroid ointment in the evening. Transitional phase: treated with 0.03% tacrolimus ointment twice daily on weekdays and concurrent application of tacrolimus and a topical corticosteroid ointment on weekend days for an additional 2 weeks. Maintenance phase: corticosteroid ointment was discontinued and 0.03% tacrolimus ointment was applied twice daily for an additional 2 weeks.	TCS, TCI, emollients	The Eczema Area and Severity Index score, Investigators’ Global Assessment, severity of pruritus and sleep disturbance scores, and QOL evaluation	None reported	Eczema Area and Severity Index scores decreased by the sixth week, and continued improvement was observed during an additional 6-week period. Both the pruritus and sleep disturbance scores decreased throughout the study. Of patients, 90% showed marked clinical improvement at week 6 and 96% at week 12.	A fixed sequential regimen of application of tacrolimus ointment with tapering of topical corticosteroids may limit the long-term use and adverse effects of topical corticosteroids, while maintaining clinical control of pediatric atopic dermatitis and improving the QOL
Keaney et al. [17]	Open-label, single-center, proof-of-concept study. Clearing phase: oral tacrolimus as monotherapy for 3 weeks. Transition phase: Oral tacrolimus (0.08 mg/ kg/d) divided in twice-daily doses + Topical Tacrolimus 0.1% ointment BID Maintenance phase: Topical Tacrolimus 0.1% ointment BID	0.03% tacrolimus ointment, oral tacrolimus	Eczema Area and Severity Index and the Physician Global Assessment scores as the primary end points.	One patient was required to discontinue oral tacrolimus because of elevations in serum creatinine and uric acid.	One patient was required to discontinue oral tacrolimus because of elevations in serum creatinine and uric acid.	Sequential therapy with oral tacrolimus and topical tacrolimus may be an effective treatment for AD. A large, randomized control study is warranted.

Patient characteristics: A total of 101 patients with AD were evaluated in four studies. The demographic characteristics of the patients in the included studies are shown in Table 2.

**Table 2:** Demographic characteristics of patients AD: atopic dermatitis

Study name	Sample size	Male/Female	Age	Disease duration/severity	Comorbidities
Takeuchi S et al. [14]	Recruited (n=70), Analyzed (n=42)	M=37, F=33; M=21, F=21	Mean (SD): 30.7 (12.5), 31.3 (13.3) 10∼24 years: 26/15	Not mentioned	Asthma: 20/12 Allergic rhinitis: 28/16
Nakahara T et al. [15]	N=17	Males, 8; females, 9	Mean age: 30.2 ± 14.2	Moderate severity with a chronic clinical course and had been treated with occasional topical corticosteroids	Not mentioned
Kubota Y et al. [16]	N=28	18/13	7.1±4.2 (range: 2-15)	3.8±3.3 (0.1-13) Mild/moderate/severe 2/25/4	Not mentioned
Keaney T et al. [17]	N=12	Not mentioned	18 years of age or older.	Severe AD covering at least 50% body surface area.	Not mentioned

Treatment characteristics: The clinical efficacy of treatment was measured using the eruption score (Nakahara et al*. *[15]) and SCORAD (Takeuchi et al*. *[14]). A detailed description of the interventions used in the four studies is provided in Table 2.

Outcome assessments: The included studies reported the data of patient improvement in terms of the Eczema Area and Severity Index, Physician Global Assessment, pruritus scores, eruption score, sleep disturbance score, quality of life, and adverse effects emerging during the course of treatment.

Overall adverse events: Serious side effects and laboratory abnormalities were absent in any of the patients whose symptoms were not severe enough to withdraw them from any of the included studies. The most common side effects were gastrointestinal tract symptoms (nausea and vomiting) following oral tacrolimus ingestion. In one patient (Kubota et al.* *[16]), oral tacrolimus was discontinued after three weeks because of a 38% elevation in serum creatinine levels and a 7% increase in serum uric acid levels from baseline. The serum creatinine level normalized in one day after discontinuation of oral tacrolimus, but the serum uric acid level remained elevated (2% increase from baseline). This patient had a baseline mild, idiopathic elevation in serum uric acid of 8.5 mg/dL (normal range 2.4-8.2 mg/dL). Mild burning sensation with tacrolimus application in one patient (Nakahara et al*. *[15]) and a transient burning sensation by topical tacrolimus, the only distinguished side effect, were recorded in 32 of 69 patients (46.3%, excluding one dropout patient who never returned after initial registration) in the induction therapy (Takeuchi et al*. *[14]). The other minor side effect was acne/folliculitis (four cases in total from the four studies combined).

Risk of Bias Assessments

**Figure 2:**
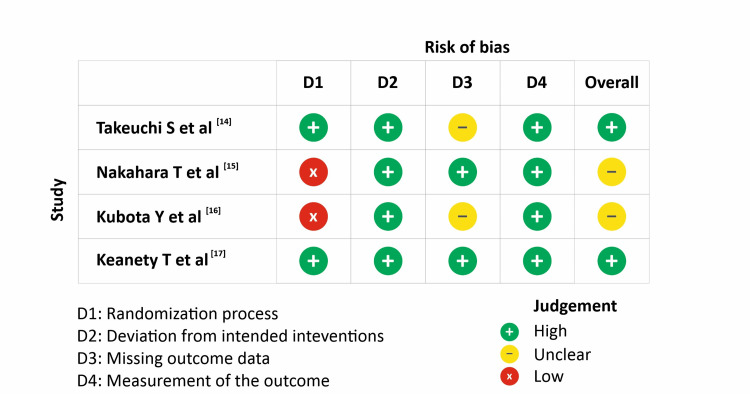
Risk of Bias assessments Takeuchi et al. [14]; Nakahara et al. [15]; Kubota et al. [16]; Keaney et al. [17]

Across the four domains considered, the overall risk of bias was low in two studies (Takeuchi et al*. *[14] and Keaney et al*. *[17]), whereas the other two had an unclear risk of bias (Nakahara et al*. *[15] and Kubota et al*. *[16]) as shown in Figure 2.

Meta-Analysis

**Figure 3:**
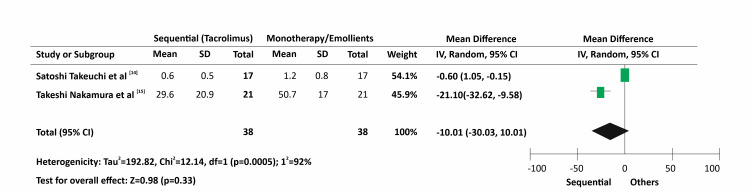
Evaluation of the efficacy of the sequential/intermittent therapy in the treatment of atopic dermatitis Takeuchi et al. [14]; Nakahara et al. [15]

A forest plot was constructed for the two studies that estimated the effectiveness of sequential therapy (Figure 3). From Figure 3, it is concluded that the study by Takeuchi et al.* *[14] is weighed higher and does not favour any of the treatment arms (sequential or otherwise). The second study by Nakahara et al*. *[15] although favouring the sequential therapy arm, weighed lower. Comparing the consistency between the trials, the two trials were found to be highly heterogeneous, as indicated by a very high I^2^ of 92% and an extremely significant p-value. The pooled estimates for sequential therapy vs. monotherapy in patients was evaluated using the Mantel Haenzal random effects model, which gave us a p-value of 0.33 with a Z value of 0.98, along with a CI of -30.03 to 10.01. The p-value was not statistically significant; however, clinically, pruritus score reduction was more evident in the sequential therapy arm than in the monotherapy arm. Takeuchi et al*. *[14] used the SCORAD system to evaluate pruritus severity and demonstrated a mean score of 0.6 ± 0.5 in the sequential therapy arm as opposed to the score of 1.2 ± 0.8, as seen in the monotherapy arm. Likewise, the eruption score seen in the study by Nakahara et al*. *[15] showed a mean score of 29.6 ± 20.9 and 50.7 ± 17 in the sequential therapy and the monotherapy arms respectively, demonstrating a clear trend favouring the sequential usage of the TCI and TCS in AD patients. Overall, pooled data from the two RCTs indicated that sequential therapy with TCS/TCI was comparable to monotherapy or emollients, as the test for overall effect determined was non-significant with a p-value of 0.33.

Discussion

AD is difficult to treat because of its multifactorial aetiology and recurrent flares that need to be kept under control for longer remission period [18]. The disease displays a high heterogeneity in its natural course and individual trajectories are unpredictable [19]; consequently the type of intervention used to prevent flares (a good preventative treatment plan) should vary depending on the individual, frequency, severity, and the sites affected by the disease. Two approaches for controlling flares in AD are proactive and reactive. Long-term, intermittent application of TCS or TCIs to previously and newly affected areas of the skin, along with maintenance treatment with emollients to unaffected areas, is a proactive approach [9]. Because of the troublesome and chronic nature of the disease, adverse effects are the main concern for the patients applying TCS for long term, the incidence and intensity of which could be reduced with the inclusion of TCIs in the regimen [20]. This warrants the inclusion of treatment regimens that not only target the lesions in patients with AD but also have an acceptable safety profile. The present study analyzed the trials involving intermittent TCS and tacrolimus sequential therapy as a proactive approach to flare and relapse control in AD treatment.

We included studies that evaluated the role of sequential/intermittent therapy with TCIs, corticosteroids, or tacrolimus. Although the sequential and intermittent use of TCI/TCS has not been clearly defined in patients with AD, we included studies that used these drugs consecutively one after the other in a phasic manner. However, only two studies satisfied this criterion and were included in the pooled analysis. The results showed a clinically significant reduction in, as evidenced by the SCORAD and the eruption scores across different studies. However, no statistically significant reduction was observed in any of the groups at the end of the treatment.

A paucity of guidelines and literature demands that sequential therapy needs to be standardized for use in AD patients. Decreased adverse effects and improved clinical outcomes are two important benefits of sequential/intermittent dual therapy for AD. In addition to providing improved clinical benefits with an acceptable safety profile, sequential therapy is associated with improved quality of life in patients with AD. According to the study findings reported by Kubota et al., the Children’s Dermatology Quality of Life Index (CDQLI) improved significantly at the end of treatment with sequential therapy [16]. Hence, sequential therapy ensures prompt clinical benefit in AD patients with a positive influence on the overall patients’ quality of life. Few researchers have evaluated the efficacy and clinical benefits of the sequential introduction of TCI and TCS in patients with AD and have highlighted its benefits in such patients. However, more studies are required to confirm the efficacy and safety of sequential therapy in patients with AD, with robust clinical and safety outcomes.

## Conclusions

Sequential therapy with topical corticosteroids and topical calcineurin inhibitors was effective and well tolerated in four clinical studies of participants with atopic dermatitis. Hence, it may be considered an important treatment approach for AD management during the initial period. 

This review aims to summarize current knowledge on the management of AD flares since their prevention is a key aim of long-term disease control; employing sequential/intermittent TCS and TCI. Inter-observer differences in AD severity ratings, limited sample size, treatment durations, and variation among patient populations included in these studies may make the results generalizable to a lesser extent to real-world scenario. Further multi-centric, global clinical trials using this approach are warranted.
